# Shoulder complaints and incidence of shoulder pathologies after contralateral major amputation in the mid and long-term

**DOI:** 10.1007/s00402-022-04720-x

**Published:** 2022-12-06

**Authors:** Valentin Rausch, Maximilian Heider, Christoph Heute, Thomas Rosteius, Dominik Seybold, Jan Geßmann, Thomas A. Schildhauer, Matthias Königshausen

**Affiliations:** 1 Department of General and Trauma Surgery, BG University Hospital Bergmannsheil, Bürkle-de-La-Camp-Platz 1, 44789 Bochum, Germany; 2 Faculty of Medicine and University Hospital, Center for Orthopedic and Trauma Surgery, University of Cologne, Kerpener Str. 62, 50937 Cologne, Germany; 3 Institute for Diagnostic and Interventional Radiology, BG University Hospital Bergmannsheil, Bochum, Germany

**Keywords:** Shoulder, Amputation, Overuse, Rotator cuff, Arthrosis

## Abstract

**Introduction:**

Amputations of the upper extremity are rare but present a life-altering event that is accompanied with considerable restrictions for the affected patients. Even with functional prosthesis, tasks of the amputated limb are usually transferred to the unaffected arm which could result in complaints of the unaffected shoulder in the mid and long term. We therefore aimed to investigate musculoskeletal pain and morphological degenerative changes of the shoulder following a contralateral amputation.

**Materials and methods:**

We included all patients with a major amputation treated at our institution with a minimum of three years since the amputation. All patients received an MRI of both shoulders and were investigated using validated scores for the upper extremity and physical activity (SSV, ASES, DASH, GPAQ, SF-36). Results of the MRIs were investigated for morphological changes by two blinded investigators comparing the side of the amputation and the unharmed upper extremity and results were correlated to the time since amputation and their physical activity.

**Results:**

A total of 20 patients with a mean age of 56 ± 19.9 years (range, 23–82 years) could be included in the study. The mean time since the amputation was 26.3 ± 19 years (range, 3–73 years). On the unharmed upper extremity, the mean SSV was 61.9 ± 24.6, the mean ASES-Score 54.5 ± 20.3, the Constant-score of 63.7 ± 40.4 and a DASH-score of 47.6 ± 23.8. The MRI of the unharmed shoulder showed significant more full-thickness rotator cuff tears and joint effusion compared to the side of the amputation. Significant differences in the degree of a glenohumeral arthritis, AC-joint arthritis, or partial rotator cuff tears could not be found between shoulders.

**Conclusion:**

Amputations of the upper extremity are associated with a high disability of the unharmed upper extremity and more full thickness rotator cuff tears compared to the side of the amputation. However, the small number of patients and rotator cuff injuries should be kept in mind when interpreting the data.

**Level of evidence:**

IV (retrospective case series).

## Introduction

Injuries resulting in an amputation of the upper limb represent only a small part of all amputations. Only 8–12% of all amputations in the USA account for upper-limb amputations. [1] In contrast to the lower limb, however, traumatic incidents are the main cause of amputations of the upper limb. [1] Those injuries result in a high degree of disability since the functions of the amputated limb are highly limited even when using active prostheses. [2] Therefore, most functions of the amputated limb are transferred to the other arm. The added functions to compensate the missing limb require extensive motions of the other arm which can result in musculoskeletal pain. [3–7] Besides the psychological complaints and disability due to the amputation, musculoskeletal pain on the other upper limb can further impair those patients. Few studies reported of musculoskeletal complaints after an upper limb amputation using questionnaires on a nationwide scale with a maximum of 108 amputees included. [3, 5–8] The aim of our study was to investigate the musculoskeletal complaints after an upper limb amputation using validated clinical scores for the upper extremity and to quantify morphological changes of the shoulder than can be attributed to the overuse in these patients. We hypothesized that after an amputation of the upper limb, patients show significant more degenerative changes and injuries to the rotator cuff compared to their contralateral shoulder.

## Material & methods

Patients receiving further treatment at our institution between 02/2007 and 02/2017 following a traumatic amputation of the upper limb with an age > 18 years were included in our study. Exclusion criteria were an injury or disease of the contralateral upper extremity unrelated to the amputation injury, less than three years since the amputation, and contraindications for a magnetic resonance imaging (MRI) of the shoulder. We chose a minimum of three years to account for adaption to the missing extremity and a possible prolonged time it can take for the fitting of a prosthesis.

All patients were examined along a standardized questionnaire including subjective and objective scores of the upper extremity: the American Shoulder and Elbow Surgeons Standardized Shoulder Assessment Form (ASES), the Subjective Shoulder Value (SSV), the Disabilities of the arm, shoulder and hand questionnaire (DASH), the Constant Score (CS), and the SF-36 questionnaire. Scores for the examination of the shoulder function were focused on the contralateral side of the amputation whenever possible, such as questions or investigations of the shoulders’ strength or range of motion. To quantify and compare the physical activity of our patients, we used the Global Physical Activity Questionnaire (GPAQ) that calculates a ‘metabolic equivalent’ by counting the total minutes of physical activity per week that are weighed according to the activity for each patient. [9]

Then, an MRI (Siemens, Magnetom Symphony 1,5 T) of both shoulder joints was acquired in all patients. We used a standard shoulder protocol (transversal and coronal PD TSE with fat saturation, coronal T_1_ TSE and sagittal T_2_ TSE). All MRIs were then interpreted by (a) an experienced radiologist and (b) an orthopedic surgeon with shoulder and elbow specialization along a standardized interpretation sheet. In case of a disagreement between the two raters, a third rater was consulted to decide, which was also an orthopedic surgeon with shoulder and elbow specialization. All raters were blinded to the side of the amputation. MRIs were particularly investigated for partial and full-thickness rotator cuff tears (RCTs), omarthrosis (classified according to Samilson and Prieto [10]), effusion and hypotrophy of the deltoid muscle, and arthrosis of the acromioclavicular joint.

Statistical analysis of the data was performed with use of SPSS (IBM SPSS Statistics, Version 25.0). To quantify the inter-rater reliability between MRI-raters, Cohens Kappa was calculated. Differences in dichotomous variables were calculated using the binominal test. Differences in classifications in MRIs were calculated using the Mann–Whitney-*U* test. To calculate the correlation of the time since the amputation with degenerative changes, the Eta-coefficient and Spearman-correlation were used, respectively. Differences in the results of the questionnaires were also calculated using Spearman-correlation. The study was reviewed and approved by the local IRB-committee (Reg.-nr.: 17-6169).

## Results

**Fig. 1:**
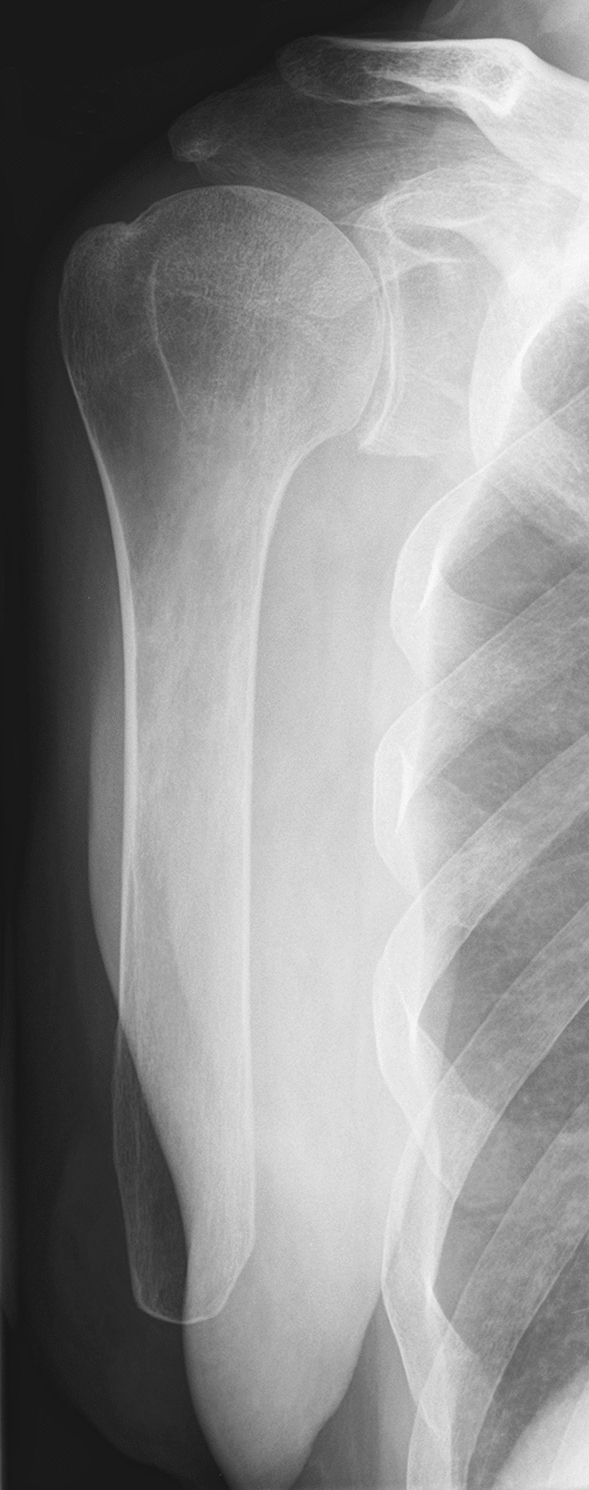
Radiographic example of a upper limb amputation (transhumeral) following a motor vehicle accident

After application of our exclusion criteria, a total of 37 patients could be included in our study. Nine patients did not want to participate in our study, 5 patients had deceased at the time of the investigation, 3 patients could not be reached for a further investigation, leaving a total of 20 patients with a mean age of 56.2 ± 16.8 years (range, 23–82 years) for the final investigation (Table 1). The amputation was a mean of 26.3 ± 18.5 (range, 3–73 years) before our investigation. In 95% (*n* = 19) a trauma was the cause of the amputation. From those, two patients were amputated after injuries due to a gunshot wound or explosion, whereas the remainder were accidents in traffic (*n* = 5), mining (*n* = 4) or other accidents at work (*n* = 8). Patients used a myoelectric prosthesis in 35%, while 40% of the patients didn’t use a prosthesis at all. Twenty-five percent of the patients only used cosmetic prosthesis (Fig. 1).

**Table 1:** Clinical data of patients

Sex: female (*n*, %)	3 (15%)
Age (mean ± SD)	56.2 ± 16.8
Age cohorts (*n*, %)	
≤ 40	3 (15%)
41–55	6 (30%)
56–70	7 (35%)
> 70	4 (20%)
Side of amputation: right (*n*, %)	12 (40%)
Level of amputation (*n*, %)	
Above elbow	11 (55%)
Elbow	1 (5%)
Forearm	7 (35%)
Wrist	1 (5%)
Prosthesis (*n*, %)	
No prosthesis	8 (40%)
Passive prosthesis	5 (25%)
Myoelectric prosthesis	7 (35%)

**Fig. 2:**
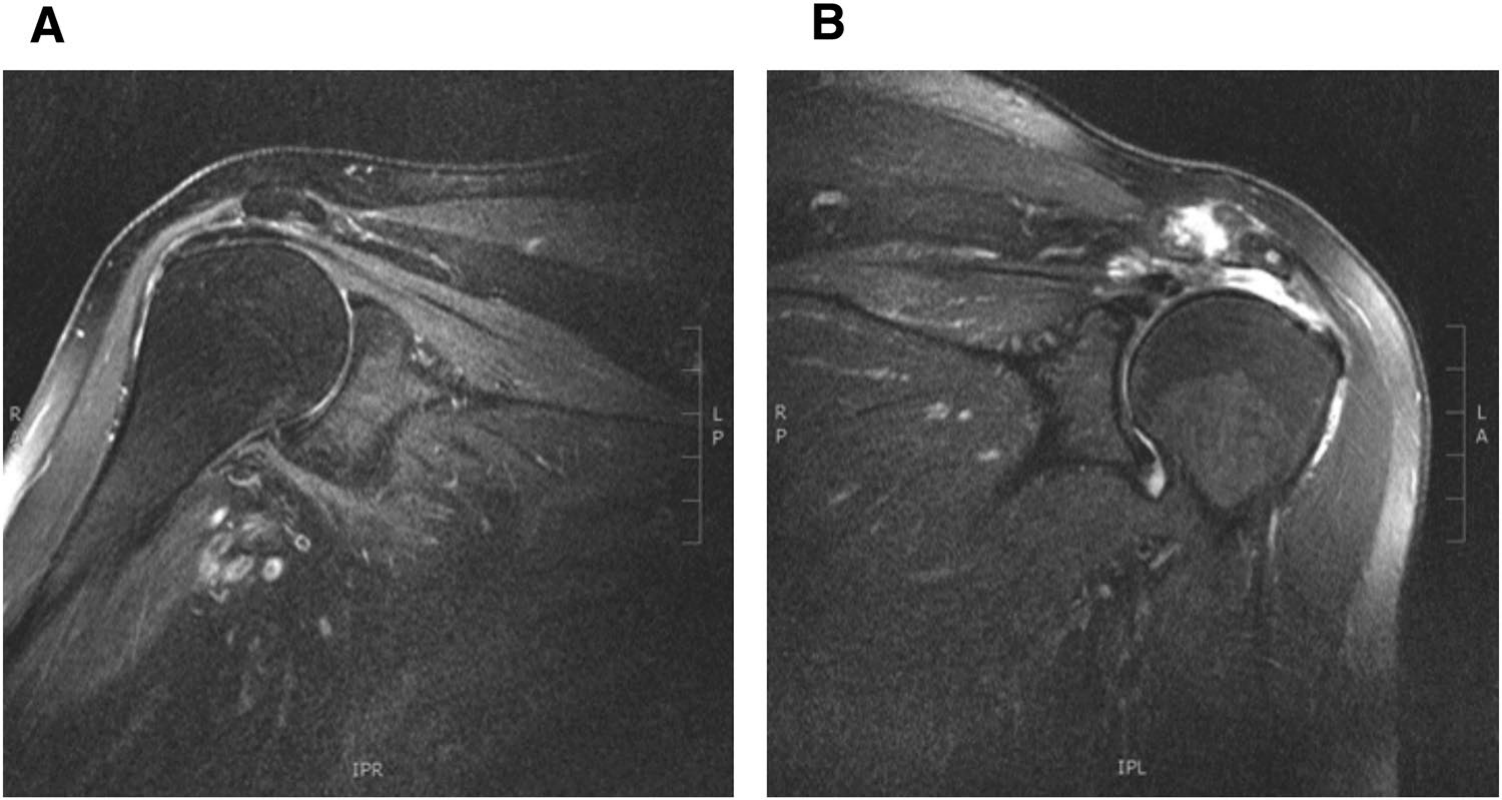
MRI-example of sagittal T2-images of a (**A**) right and (**B**) left shoulder. This patient suffered an upper limb amputation of the right side. Note the full-thickness rotator cuff tear besides other degenerative changes of the shoulder joint

In the shoulder-MRIs, patients showed significant more full-thickness RCTs (*p* < 0.05) and intraarticular effusions on the contralateral side of the amputation (*p* < 0.001) as well as a deltoid atrophy on the amputated side (*p* < 0.001) (Fig. 2). No significant differences could be shown regarding partial RCTs or omarthrosis (Table 2). An overview of MRI-findings in each subject can be found in Table 3*.*

**Table 2:** Results of degenerative changes and inter-rater agreement of MRI-investigations

	Amputated side (*n*, %)	Contralateral side (*n*, %)	*p*-value	Cohens Kappa
Full-thickness RCT	1 (5%)	4 (20%)	0.016	0.875
Partial RCT	14 (70%)	9 (45%)	0.1	0.648
Joint effusion	0 (0%)	2 (10%)	< 0.001	1.0
Subacromial effusion	4 (20%)	6 (30%)	0.196	0.353
Omarthrosis	3 (15%)	4 (20%)	0.485	0.924
AC-joint arthrosis	14 (70%)	15 (75%)	0.416	0.617
Deltoid hypotrophy	17 (85%)	1 (5%)	< 0.001	0.963

**Table 3:** Correlation between objective and subjective shoulder scores

	Age		Time since amputation	
	Correlation coefficient	*p*-value	Correlation coefficient	*p*-value
SSV	– 0.586	0.005	– 0.599	0.004
ASES	– 0.309	0.173	– 0.434	0.049
DASH	0.299	0.188	0.452	0.04

**Fig. 3:**
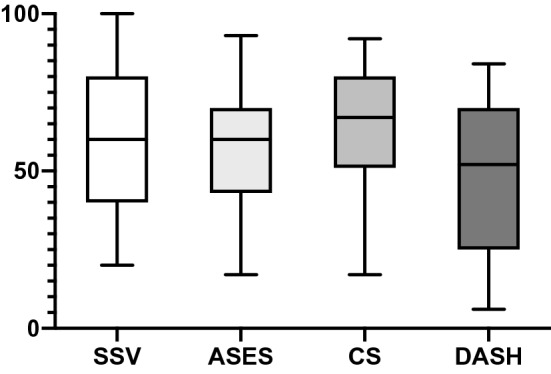
Boxplots (mean, standard deviation, and range) of objective and subjective scores

**Fig. 4:**
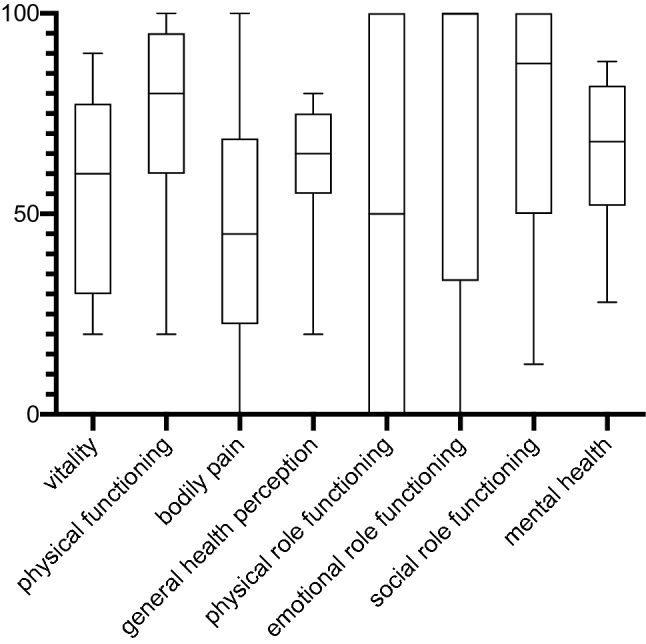
Boxplots (mean, standard deviation, and range) of SF-36 subgroups

Mean results of the functional scores are shown in Fig. 3. The largest impairment in the SF-36 score could be shown in the bodily pain section with a mean of 45.6 ± 28 (Fig. 4). All upper extremity-specific scores showed a significant impairment of the upper limb. The mean SSV reached 62 ± 24, the ASES-score 54.5 ± 19.8, the Constant Score 64 ± 20, and the DASH-score 47.6 ± 23.2. In the ASES-score, 57.1% of the patients had at least substantial complaints on their upper extremity.

The correlation of significant differences in the MRI between the shoulder of the amputated and the contralateral side (FT-RCT and joint effusion) to the age of the patient and the latency to the amputation showed a moderate correlation to both the age (eta-coefficient: 0.440) and the latency to the amputation (eta-coefficient: 0.492), whereas no significant correlation could be found for joint effusion in relation to age (eta-coefficient: 0.055) or latency to the amputation (eta-coefficient: 0.084).

When correlating the scores with the latency to the amputation and the age of the patient, both the SSV and the CS showed a significant worse result (Table 4). The DASH-score and the ASES-score showed a significant worse result in correlation to the latency of the amputation but not to the age of the patient. The type of prosthesis used by the patient did not show a significant correlation to the results of any evaluated score.

**Table 4:** Overview of MRI findings in each subject divided between the amputated side (amp.) and the contralateral side (contr.) showing full-thickness rotator cuff tears (ft-RCT), partial-thickness RCT (pt-RCT), glenohumeral osteoarthritis (GHOA), AC-joint osteoarthritis (ACJOA), joint effusion, and a side with larger deltoid atrophy (if applicable)

Age	Latency (y)		ft-RCT	pt-RCT	GHOA	ACJOA	Joint-effusion	Deltoid atrophy
41	15	Amp	No	Yes	No	No	No	×
		Contr	No	Yes	No	No	No	
59	16	Amp	No	Yes	No	Yes	No	×
		Contr	No	Yes	No	No	No	
67	41	Amp	No	Yes	No	No	No	×
		Contr	Yes	N/A	No	Yes	No	
31	15	Amp	No	No	Yes, S/P 1	Yes	No	×
		Contr	No	No	Yes, S/P 1	No	No	
54	27	Amp	No	No	No	Yes	No	N/A
		Contr	No	No	No	No	No	
76	28	Amp	No	Yes	Yes, S/P 3	Yes	No	N/A
		Contr	Yes	N/A	Yes, S/P 2	Yes	No	
70	34	Amp	No	Yes	No	Yes	No	×
		Contr	No	Yes	No	Yes	No	
53	6	Amp	No	Yes	No	Yes	No	×
		Contr	No	No	No	Yes	No	
58	9	Amp	No	No	No	Yes	No	×
		Contr	No	Yes	No	Yes	No	
59	5	Amp	No	Yes	Yes, S/P 2	Yes	No	×
		Contr	No	Yes	Yes, S/P 3	Yes	Yes	
23	13	Amp	No	No	No	No	No	×
		Contr	No	No	No	yes	No	
47	25	Amp	No	Yes	No	Yes	No	×
		Contr	No	No	No	Yes	No	
82	73	Amp	No	Yes	No	Yes	No	×
		Contr	Yes	N/A	Yes, S/P 1	Yes	No	
44	3	Amp	No	Yes	No	No	No	×
		Contr	No	No	No	Yes	No	
65	35	Amp	No	Yes	No	Yes	No	x
		Contr	No	No	No	Yes	No	
59	38	Amp	No	Yes	No	No	No	×
		Contr	Yes	N/A	No	Yes	Yes	
27	3	Amp	No	Yes	No	No	No	×
		Contr	No	Yes	No	No	No	
79	56	Amp	Yes	N/A	No	Yes	No	×
		Contr	No	yes	No	Yes	No	
50	28	Amp	No	No	No	Yes	No	×
		Contr	No	Yes	Yes, S/P 1	Yes	No	
80	56	Amp	No	Yes	No	Yes	No	
		Contr	No	Yes	No	Yes	No	×

When correlating the metabolic equivalent of the GPAQ as a measurement of physical activity to the shoulder function measured with the use of the ASES score, a moderate positive correlation could be found in our collective (r: 0.449, *p*-value: 0.047).

## Discussion

An amputation of an upper extremity is a life-altering event that results in a high prevalence of musculoskeletal pain and impaired mental health. [6] In our study, those patients experience impairment of their upper extremity, show significant more full-thickness RCTs in the contralateral shoulder, regularly suffer from bodily pain, and showed exceptional poor clinical scores of the upper extremity compared to a normal population in the respective age [11–13].

One reason for the high prevalence of musculoskeletal pain in these patients is that functions of the amputated limb are usually transferred to the remaining arm. This can partially be explained with the limited functionality of upper-limb prostheses. In contrast to protheses of the lower limb, even more functional myoelectrical prosthesis of the upper limb are barely able to compensate the missing functions of the upper limb. [2] As a result, most of the more complex tasks of the upper limb are transferred to the unaffected side even though use of myoelectrical prosthesis could be shown as a prevention for musculoskeletal complaints of the unaffected side. [7, 14] However, in our study, only 60% of the patients did use a prosthesis on a regular basis which is in line with previous published percentages. [15, 16]

Transfer of tasks to the unaffected arm results in a higher prevalence of degenerative changes to the unaffected arm. These changes due to an overuse of the unaffected side were discussed by Østlie et al., who suspected symptomatic rotator cuff injuries significantly more on the unaffected side after an upper-limb amputation. [17] Comparable morphological changes are also well-known in other patients with an overuse of the upper limb such as paraplegic patients or overhead-athletes in wheelchairs. [18, 19] Rotator cuff tears are commonly degenerative changes of the shoulder that were overall low in our study although we could find considerable more FT-RCTs in the contralateral shoulder of the amputation. However, similar rates of full thickness RCTs as a surrogat for degenerative can be found in the general population without an amputation of one upper limb. [20, 21] In contrast, partial thickness rotator cuff tears were not found to be significantly more frequent in one shoulder. We believe the higher absolute number of partial thickness rotator cuff tears in the amputated site that did not reach significance was most likely due to the lower diagnostic accuracy of these injuries and lies within the margin of error. Significantly lower degenerative changes to the shoulder joint and a hypotrophy of the deltoid muscle on the amputated side as shown in our study can be explained by underuse of this joint. In addition, we could find a moderate correlation between full-thickness RCTs and the time since the amputation, with a similar correlation to the age of the patients.

In this study we could find considerable impairments in objective and subjective shoulder scores as well as overall substantial degenerative changes to the unaffected shoulder after an amputation of the upper limb. Another interesting notion is the highly significant hypotrophy of the deltoid muscle on the amputated side, underlining the underuse of this muscle. This could indicate that even the use of an upper limb prosthesis does not result in a relevant use of this muscle in everyday life. Besides the use of (functional myoelectrical) prostheses of the arm, prevention might reduce musculoskeletal complaints of the upper limb due to overuse after an amputation. Education regarding the awareness of early symptoms and following therapies could present a possibility to reduce musculoskeletal complaints in these patients. Physical exercise could also help prevent these complaints as it is supported by a negative correlation of a higher physical activity and a better function of the unaffected shoulder in our study. Nonetheless the higher physical activity of the patients could also be due to the less musculoskeletal complaints in these patients.

Our study has certain limitations. In general, only 20 patients could be included in our study due to the rarity of upper limb amputations. Larger studies in a multicentric design are necessary to test these findings. Another limitation arises from the missing control group for degenerative changes of the shoulder and the clinical scores. We used the contralateral shoulder to detect differences in degenerative changes. However, since the amputated upper limb has a significant less risk of overuse, higher rates of degenerative changes could also be due to degeneration that is also present in the general population. Overall clinical scores however showed exceptional poor outcomes even without a matching control cohort.

## Conclusion

Patients suffer high rates of musculoskeletal pain and an impaired shoulder function on the contralateral side after an upper-limb amputation. Moreover, compared to the amputated side, more full-thickness rotator cuff changes can be found.

## Data Availability

The data that support the findings of this study are available from the corresponding author, Valentin Rausch, upon reasonable request.
